# Reducing YAP expression in *Pkd1* mutant mice does not improve the cystic phenotype

**DOI:** 10.1111/jcmm.15512

**Published:** 2020-06-27

**Authors:** Chiara Formica, Sandra Kunnen, Johannes G. Dauwerse, Adam E. Mullick, Kyra L. Dijkstra, Marion Scharpfenecker, Dorien J. M. Peters, the DIPAK Consortium

**Affiliations:** ^1^ Department of Human Genetics Leiden University Medical Center Leiden The Netherlands; ^2^ IONIS Pharmaceuticals Carlsbad California USA; ^3^ Department of Pathology Leiden University Medical Center Leiden The Netherlands

**Keywords:** 3D cysts, ADPKD, ASO, Hippo pathway, Yap/Taz

## Abstract

The Hippo pathway is a highly conserved signalling route involved in organ size regulation. The final effectors of this pathway are two transcriptional coactivators, yes‐associated protein (YAP) and transcriptional coactivator with PDZ‐binding motif (WWTR1 or TAZ). Previously, we showed aberrant activation of the Hippo pathway in autosomal‐dominant polycystic kidney disease (ADPKD), suggesting that YAP/TAZ might play a role in disease progression. Using antisense oligonucleotides (ASOs) in a mouse model for ADPKD, we efficiently down‐regulated *Yap* levels in the kidneys. However, we did not see any effect on cyst formation or growth. Moreover, the expression of YAP/TAZ downstream targets was not changed, while WNT and TGF‐β pathways' downstream targets *Myc*, *Acta2* and *Vim* were more expressed after *Yap* knockdown. Overall, our data indicate that reducing YAP levels is not a viable strategy to modulate PKD progression.

## 1 INTRODUCTION

The Hippo pathway is a highly conserved signalling route involved in the regulation of key cellular processes like proliferation, apoptosis and differentiation, which ultimately results in the regulation of organ size. When the Hippo pathway is inactive, the pathway effectors yes‐associated protein (YAP) and its paralogue transcriptional coactivator with PDZ‐binding motif (WWTR1 or TAZ) are unphosphorylated and can shuttle to the nucleus where they can drive the transcription of genes involved in proliferation and apoptosis.^1^, ^2^

In a previous study, we showed altered Hippo signalling in autosomal‐dominant polycystic kidney disease (ADPKD).^3^ ADPKD is characterized by progressive deterioration of kidney function as a consequence of the formation of thousands of epithelium‐derived cysts, leading to renal failure beyond mid‐life. In the majority of the cases, ADPKD is caused by a mutation in either the *PKD1* or *PKD2* gene, which encodes for polycystin1 (PC1) and polycystin2 (PC2), respectively.^4^, ^5^ We observed strong nuclear accumulation of YAP in dilated tubules and cysts of several orthologous mouse models, as well as in human ADPKD cystic kidneys and cystic liver tissues.^3^ Therefore, we hypothesize that reducing YAP levels using antisense oligonucleotides (ASOs) may slow down the cystic renal disease in iKsp*Pkd1* ^del^ mice.

## 2 MATERIALS AND METHODS

### 2.1 Experimental animals and study design

All the animal experiments performed have been approved by the local animal experimental committee of the Leiden University Medical Center and the Commission Biotechnology in Animals of the Dutch Ministry of Agriculture.

Inducible kidney‐specific *Pkd1* deletion mice (iKsp*Pkd1* ^del^) and tamoxifen administration have been described before.^6^ *Pkd1* gene has been knocked out at post‐natal day 18 (PN18). 32 male mice have been divided into two experimental groups of 16 animals each: one received scrambled antisense oligonucleotide (ASO), and the other received *Yap*‐specific ASO. Both groups received an injection of 100 mg/kg of ASO via i.p. injection, starting two weeks after *Pkd1* inactivation (PN18 + 2 weeks), once a week, until week 7 after gene inactivation. Mice were sacrificed at 8 weeks after gene inactivation (PN18 + 8 weeks). ASOs were provided by Ionis Pharmaceuticals. Both ASOs were 16mer S‐constrained ethyl gapmers with a 3‐10‐3 chimeric design and a phosphorothioate backbone. *Yap* ASO sequence was as follows: 5′‐AACCAACTATTACTTC‐3′; scrambled ASO sequence was as follows: 5′‐ GGCCAATACGCCGTCA‐3′. The *Yap* ASO was selected from leads identified following in vitro screens which were then evaluated in vivo for renal activity and tolerability. Scrambled ASO did not bind to any known target and was included as a control for non‐specific effects.

At sacrifice, both kidneys were collected and used for immunohistochemistry (IHC) or snap‐frozen for RNA and protein extraction. Blood urea nitrogen level (BUN) was measured using the Reflotron Plus (Roche Basel). Three age‐matched wild‐type (Wt) mice were also included for IHC purposes.

### 2.2 Cell culture

Wt mouse inner medulla collecting duct cells from ATCC (mIMCD3, CRL‐2123™ ATCC^®^) and Madin‐Darby canine kidney (MDCK) cells (CCL‐34™; ATCC) were commercially available. Briefly, cells were maintained at 37°C, and 5% CO_2_ in DMEM/F‐12 with GlutaMAX (#31331‐093; Gibco, Life Technologies) supplemented with 100 U/mL penicillin‐streptomycin (#15140‐122; Gibco, Life Technologies) and 10% foetal bovine serum (#S1860; Biowest). Cell cultures were monthly tested for mycoplasma contamination using MycoAlert Mycoplasma Detection Kit.

For 3D cyst assay, cells were grown in Matrigel as described previously.^7^ Briefly, cells were mixed with Matrigel (#354230) supplemented with 10% rat tail collagen I (kindly provided by OcellO BV) and seeded in 96‐wells. Cells were cultured in normal condition for 72 hours and subsequently stimulated with forskolin (#344270, Calbiochem, Millipore BV) or DMSO for 72 hours. Cells were collected for immunohistochemistry (IHC).

Generation of the Pkd1 knockout cell line mIMRFNPKD 5E4 using the dimeric CRISPR RNA‐guided FokI nuclease (RFN) method was described before.^7^ A comparable method was used to generate the mIMCD3 *Yap1* knockout cell lines and to knockout *Yap* from the *Pkd1* knockout cell line mIMRFNPKD 5E4 to generate a *Yap*/*Pkd1* double knockout. More details available upon request.

### 2.3 Immunohistochemistry and Western blotting

For IHC, formalin‐fixed paraffin‐embedded kidneys or cysts were sectioned at 4µm thickness. Sections were stained with haematoxylin and eosin (H&E), periodic acid‐Schiff (PAS) or with these antibodies: rabbit anti‐YAP (1:800 for kidneys and 1:1000 for 3D cysts; #14074; Cell Signaling Technology); rabbit anti‐TAZ (1:500 for kidneys and 1:1000 for 3D cysts; #4883; Cell Signaling Technology); rabbit anti‐Ki‐67 (1:3000, Novocastra, Leica Biosystems); rabbit anti‐aquaporin‐2 (1:4000, Calbiochem); goat anti‐uromodulin (also known as anti‐Tamm‐Horsefall, 1:500, Organon Teknika‐Cappel); and rabbit anti‐megalin (1:500, LUMC^3^).

For Western blot, snap‐frozen kidneys were homogenized using the Magnalyser technology (Roche) in RIPA buffer supplemented with protease inhibitor cocktails (#05892970001; Roche). Antibodies used: rabbit anti‐YAP (1:1000; #14074; Cell Signaling Technology), rabbit anti‐TAZ (1:1000; #4883; Cell Signaling Technology) and mouse anti‐GAPDH (1:5000; #97166; Cell Signaling Technology). Secondary antibodies: goat anti‐rabbit IRDye 800CW (1:10 000; #926‐32211; LI‐COR Biosciences; Lincoln, NE, USA) and goat anti‐mouse IRDye 680RD (1:10 000; #926‐32220; LI‐COR Biosciences).

### 2.4 Quantification of Ki‐67‐positive cells

Formalin‐fixed paraffin‐embedded kidneys were sectioned at 4 µm thickness and stained overnight at room temperature with rabbit anti‐Ki‐67 antibody and counterstained with haematoxylin. Sections were acquired using Philips Ultra Fast Scanner at 20× magnification factor, and pictures of 15 random areas of the kidney were taken. ImageJ software (public domain software, NIH) was used to measure the Ki‐67‐positive area and the haematoxylin‐positive area. The relative Ki‐67 area was calculated as a percentage of the ratio of Ki‐67‐positive area over haematoxylin‐positive area.

### 2.5 Gene expression analysis

Total RNA was isolated from snap‐frozen kidneys using TRI Reagent (#T9424; Sigma‐Aldrich) according to manufacturer's protocol, and gene expression analysis was performed by quantitative PCR (qPCR) as described previously.^8^ Briefly, cDNA synthesis was done using Transcriptor First Strand cDNA Synthesis Kit (#04897030001; Roche) according to the manufacturer's protocol. qPCR was done in triplicate on the LightCycler 480 II (Roche) using 2× FastStart SYBR‐Green Master (#04913914001; Roche) according to the manufacturer's protocol. Data were analysed with LightCycler 480 Software, Version 1.5 (Roche). Gene expression was normalized to the housekeeping gene *Hprt*. Primer sequences available upon request.

### 2.6 Statistical analysis

Data were analysed using GraphPad Prism 7.00 for Windows (GraphPad Software, www.graphpad.com).

## 3 RESULTS

### 3.1 YAP knockdown using ASOs does not improve cystic phenotype in vivo

We showed in the past that cyst‐lining epithelia have intense nuclear YAP localization, both in *Pkd1‐*mutant mouse models and in ADPKD patients.^3^ Therefore, we hypothesized that YAP could actively contribute to cyst formation or cyst growth, through up‐regulation of target genes involved in cell proliferation and apoptosis.

**Figure 1:**
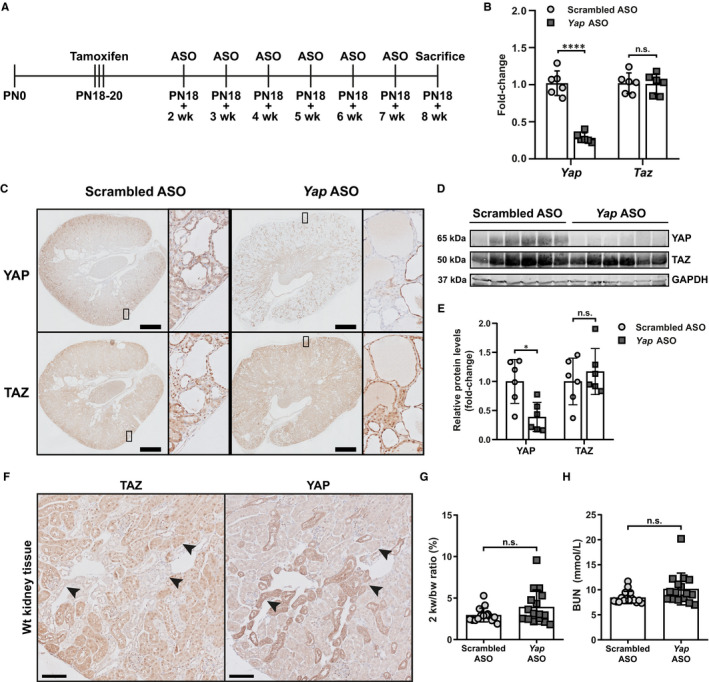
In vivo down‐regulation of *Yap* with ASOs. A, Schematic representation of the in vivo experimental pipeline. *Pkd1* gene inactivation was achieved with three consecutive administrations of tamoxifen at post‐natal day 18 (PN18). Two weeks after gene inactivation, mice were injected weekly intraperitoneally with *Yap*‐specific ASO or scrambled ASO as control. The last ASO injection was performed at 7 weeks after gene inactivation, and the mice were sacrificed one week later (+8 wk). B, Gene expression (fold change) of *Yap* and *Taz* at the sacrifice in mice treated with *Yap* ASO and scrambled ASO. Each symbol represents a mouse. Mean with ± SD. **** *P* < .0001, *t*‐test. C, Representative IHC of renal tissue from mice treated with scrambled ASO and *Yap* ASO, showing YAP and TAZ. Scale bar 1 mm. D, Total kidney protein lysates of mice treated with scrambled ASO and *Yap* ASO blotted for endogenous YAP, TAZ and GAPDH. E, Quantification of YAP and TAZ protein level in total kidney normalized on GAPDH. Each symbol represents a mouse. Mean with ± SD. * *P* < .05, n.s. not significant, *t*‐test. F, Representative YAP and TAZ IHC on sequential slides of Wt mice kidneys at post‐natal day 100. Arrowheads show the same tubules stained for the two different proteins. Scale bar 200 µm. G, Quantification of kidney size using two kidney weight/bodyweight ratio. n.s. not significant. H, Blood urea nitrogen (BUN) level at the sacrifice. n.s. not significant

To check the effect of YAP on the cystic phenotype in vivo, we knocked down *Yap* using ASOs in young adult iKsp*Pkd1* ^del^ mice. The *Pkd1* gene was inactivated in 18‐day‐old mice (PN18), and 2 weeks after gene inactivation they were injected via i.p., with *Yap*‐specific ASO (n = 16) or scrambled ASO (n = 16), every week until sacrifice. Mice were sacrificed 8 weeks after gene inactivation (PN18 + 8 weeks) (Figure 1A).

The *Yap* ASO treatment resulted in about 70% reduction of *Yap* gene expression levels without affecting *Taz* expression, confirming its efficacy and specificity in vivo (Figure 1B). YAP reduction was confirmed at the protein level, while TAZ protein expression was unchanged by the ASO treatment (Figure 1C‐E). Interestingly, we observed a distinct pattern of expression for YAP and TAZ in kidneys, with tubule segments strongly positive for one protein but showing low expression for the other. This is even clearer in Wt mice, where the architecture of the kidney is intact (Figure 1F). Thus, YAP and TAZ importance in the homeostasis of the various renal segments might be different.

**Figure 2:**
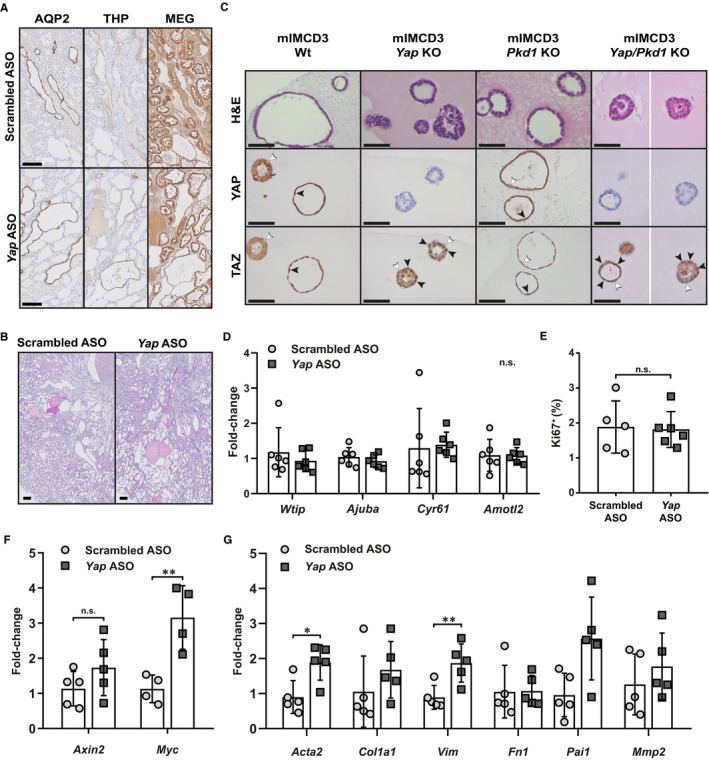
Effect of YAP modulation in vivo and in 3D cultures (A) Representative IHC for aquaporin‐2 (AQP2—collecting duct), Tamm‐Horsfall (THP—distal tubules) and megalin (MEG—proximal tubules) on sequential slides of ASO‐treated mice kidneys. The staining shows the presence of dilated tubules and small cysts that are positive for each marker. Scale bar 100 µm. B, Representative periodic acid‐Schiff (PAS) staining of renal tissue from mice treated with scrambled ASO and *Yap* ASO. Scale bar 200 µm. C, H&E staining of formalin‐fixed, paraffin‐embedded cysts grown in Matrigel after forskolin stimulation (top row). Representative IHC of formalin‐fixed, paraffin‐embedded cysts of forskolin‐treated Wt and mutant mIMCD3 cells stained for YAP (middle row) and TAZ (bottom row). Wild‐type epithelial cells and *Pkd1* KO cells grown in Matrigel spontaneously develop cystic structures with a visible lumen and can swell after forskolin stimulation. *Yap* KO cells and *Yap*/*Pkd1* double KO cells grown in Matrigel showed impaired cyst formation, with the majority of the cells developing tumour‐like mass structures, which did not respond to forskolin stimulation. Only the sporadic cysts that developed a normal lumen before forskolin treatment increased in size after stimulation. For mIMCD3 *Yap*/*Pkd1* KO, cysts from multiple fields of view are shown separated by a white line. White arrowheads indicate cytoplasmic localization of the proteins; black arrowheads indicate nuclear localization of the proteins. Scale bar 50 µm. D, Gene expression (fold‐change) of YAP/TAZ targets at the sacrifice in mice treated with *Yap* ASO and scrambled ASO. Each symbol represents a mouse. Mean with ± SD. n.s. (not significant) refers to all the genes in the graph, *t*‐test. E, Quantification of Ki‐67‐positive area. Each symbol represents a mouse. Mean with ± SD. n.s. not significant, *t*‐test. F, Gene expression (fold change) of WNT pathway targets *Axin2* and *Myc* at the sacrifice in mice treated with *Yap* ASO and scrambled ASO. Each symbol represents a mouse. Mean with ± SD. ** *P* < .01, n.s. not significant, *t*‐test. G, Gene expression (fold‐change) of TGF‐β pathway targets, *Acta2*, *Col1a1*, *Vim*, *Fn1*, *Pai1* and *Mmp2*, at the sacrifice in mice treated with *Yap* ASO and scrambled ASO. Each symbol represents a mouse. Mean with ± SD. * *P* < .05, ** *P* < .01, *t*‐test. If no significance is indicated, the comparison is not significant

Analysis of the kidneys sizes, by measuring two kidney weight/bodyweight ratio's, and of renal function, using BUN levels, revealed comparable disease progression in the two experimental groups (Figure 1G, H). Segment‐specific IHC and PAS staining revealed tubule dilation and cyst formation in every kidney segments, both in *Yap* ASO and scrambled ASO treated mice, suggesting that *Yap* knockdown did not affect cyst formation in vivo (Figure 2A, B).

In vitro, 3D culture of *Yap* KO or *Yap/Pkd1* KO renal epithelial cells resulted in abnormal cyst formation, with only sporadic lumen formation and development of tumour‐like structures (Figure 2C). However, the sporadic cysts that are formed did not seem to show impaired growth, consistently with in vivo data. Nuclear staining of TAZ was clearly observed in *Yap* KO cells, suggesting that lack of YAP increases TAZ shuttling in vitro (Figure 2C).

### 3.2 YAP and TAZ downstream targets expression is not changed by *Yap* knockdown in vivo

YAP and TAZ are transcriptional coactivators and can translocate into the nucleus where they can drive gene expression. To study the effect of *Yap* knockdown on the expression of its target genes, we quantified the expression of known YAP/TAZ targets, *Wtip*, Ajuba, *Cyr61* and *Amotl2*.^9^ Despite the consistent *Yap* reduction at the mRNA level, the expression of target genes is not changed in *Yap* ASO‐treated compared to scrambled ASO‐treated mice (Figure 2D). Additionally, we evaluated the expression of Ki‐67, a marker for cell proliferation, as YAP and TAZ can regulate transcriptional programmes that control cell proliferation.^9^ We did not find significant differences between *Yap* ASO and scrambled ASO treated mice (Figure 2E). In conclusion, the knockdown of *Yap* does not affect the expression of the downstream targets we tested.

### 3.3 WNT and TGF‐β pathways seem to be more active in *Yap* ASO mice

It is well known that both YAP and TAZ can interact with the final effectors of the WNT and TGF‐β pathways.^10^ For this reason, we checked the expression levels of several target genes regulated by β‐catenin (*Axin2* and *Myc*) and Smads (*Acta2*, *Col1a1*, *Vim*, *Fn1*, *Pai1* and *Mmp2*). We observed increased *Myc* expression in *Yap* ASO‐treated mice compared to scrambled ASO, but only a trend for *Axin2* expression (Figure 2F). Moreover, we saw significantly increased expression of alpha smooth muscle actin (*Acta2*) and vimentin (*Vim*) and a consistent trend for collagen 1 alpha 1 (*Col1a1*), fibronectin (*Fn1*), plasminogen activator inhibitor‐1 (*Pai1*) and matrix metallopeptidase 2 (*Mmp2*) (Figure 2G). Thus, although not conclusive, these results suggest that WNT and TGF‐β pathways are more active upon *Yap* knockdown.

## 4 DISCUSSION

To investigate YAP as a potential target for therapeutic intervention in PKD, we used ASOs to selectively knockdown the expression of *Yap* in young adult iKsp*Pkd1* ^del^ mice. We reached about 70% reduction in gene expression, indicating that ASOs can be a viable strategy to effectively and selectively down‐regulate a target in kidneys in models for PKD. Our data clearly indicate that *Yap* knockdown using ASOs in a mouse model for ADPKD does not improve the cystic phenotype.

Considering that TAZ (or WWTR1) levels are not changed by *Yap* ASO, we hypothesized that TAZ could be compensating for *Yap* knockdown. Indeed, expression levels of the target genes are not changed by *Yap* knockdown, and TAZ shows a clear nuclear localization in most of the cystic and dilated renal tubules, as well as in 3D cyst culture of *Yap* KO. Moreover, double knockout of *Yap* and *Taz* in *Pkd1*‐deficient mice was able to reduce PKD progression mildly.^11^ Hence, targeting TAZ together with YAP might be a viable strategy to inhibit cyst progression. However, TAZ physically interacts with PC1 and PC2, participating in common signalling routes involved in cyst formation. Moreover, knockout of *Taz* leads to cysts formation in mice and zebrafish, even in the absence of a *Pkd1* mutation.^11^, ^12^, ^13^ Thus, further studies are needed to clarify the role of TAZ in PKD.

A recent paper showed that *Yap* KO was able to reduce PKD progression mildly in *Pkd1*‐deficient mice.^14^ However, the mouse model used and the *Yap*‐targeting strategy were different, providing a possible explanation for the dissimilar outcomes. In their study, they knocked out the different genes simultaneously, while we selectively reduced YAP expression with a strategy based on antisense oligonucleotides that could potentially be translated to the clinic.

YAP and TAZ are transcriptional coactivators that can modulate a variety of biological processes. They have been associated with fibrogenesis and epithelial‐to‐mesenchymal transition in vivo and in vitro via interaction with both TGF‐β and WNT signalling pathways. Particularly, when the Hippo pathway is active, YAP and TAZ are phosphorylated and restrained in the cytoplasm where they can interact with SMADs and β‐catenin, preventing their nuclear translocation and transcriptional activity.^10^ In our study, we observed increased expression of some of the downstream targets of WNT and TGF‐β pathways in *Yap* ASO treated mice. This might suggest that reduced YAP levels cause an imbalance in the regulation of these interacting signalling pathways, either because YAP cannot physically interact with SMADs and β‐catenin any more, or due to increased activation of TAZ overcompensating for YAP loss. As increased activation of WNT or TGF‐β pathways is well known for promoting cyst formation in ADPKD,^15^, ^16^ it might explain why we did not see any amelioration of the phenotype with *Yap* knockdown in vivo. Further studies are necessary to unveil the exact molecular mechanisms.

In conclusion, although we cannot exclude that the Hippo pathway is involved in cyst growth, we believe that the strong nuclear YAP localization observed in cyst‐lining epithelia is more a consequence of cell stretching rather than a driving force for cell proliferation. Indeed, down‐regulation of YAP using ASOs did not affect cell proliferation nor the cystic phenotype in our *Pkd1* KO mouse model. Moreover, due to its profound interconnection with other signalling pathways, such as WNT and TGF‐β, a therapeutic intervention for PKD based on the modulation of YAP levels might not be feasible, at least with the current knowledge.

## CONFLICT OF INTEREST

The authors confirm that there are no conflicts of interest.

## AUTHOR CONTRIBUTION

**Chiara Formica:** Conceptualization (equal); Data curation (lead); Formal analysis (equal); Investigation (lead); Visualization (lead); Writing‐original draft (equal). **Sandra Kunnen:** Conceptualization (equal); Data curation (equal); Formal analysis (equal); Investigation (supporting); Writing‐original draft (supporting). **Johannes G Dauwerse:** Data curation (equal); Investigation (supporting); Writing‐original draft (supporting). **Adam E Mullick:** Methodology (lead); Writing‐original draft (supporting). **Kyra L Dijkstra:** Data curation (equal); Investigation (supporting); Writing‐original draft (supporting). **Marion Scharpfenecker:** Formal analysis (equal); Investigation (supporting); Writing‐original draft (supporting). **Dorien JM Peters:** Conceptualization (equal); Formal analysis (equal); Funding acquisition (lead); Investigation (equal); Project administration (lead); Supervision (lead); Visualization (supporting); Writing‐original draft (equal).

## Data Availability

The data that support the findings of this study are available from the corresponding author upon reasonable request.
